# TWIST1 associates with NF-κB subunit RELA via carboxyl-terminal WR domain to promote cell autonomous invasion through IL8 production

**DOI:** 10.1186/1741-7007-10-73

**Published:** 2012-08-14

**Authors:** Shan Li, Stephen E Kendall, Raquel Raices, James Finlay, Maricela Covarrubias, Zheng Liu, Gina Lowe, Yu-Huey Lin, Yuan Han Teh, Victoria Leigh, Simi Dhillon, Steven Flanagan, Karen S Aboody, Carlotta A Glackin

**Affiliations:** 1 Division of Neurosciences, Beckman Research Institute of the City of Hope, Duarte, CA 91010, USA; 2 Department of Neurosurgery, Beckman Research Institute of the City of Hope, Duarte, CA 91010, USA; 3 Department of Molecular Medicine, Beckman Research Institute of the City of Hope, Duarte, CA 91010, USA; 4 Irell & Manella Graduate School of Biological Sciences, Beckman Research Institute of the City of Hope, Duarte, CA 91010, USA; 5 Department of Biological Sciences, California State Polytechnic Institute, Pomona, CA 91768, USA; 6 GeneTex, Irvine, CA 92606, USA; 7 Affymetrix, Santa Clara, CA 95051, USA; 8 Western University of Health Sciences, Pomona, CA 91766, USA; 9 University of California Berkeley, Berkeley, CA 94720, USA; 10 StemCell Technologies Inc., Vancouver, BC V5Z 1B3, Canada

**Keywords:** TWIST1, WR domain, RELA, NF-κB, IL8

## Abstract

**Background:**

Metastasis is the primary cause of death for cancer patients. TWIST1, an evolutionarily conserved basic helix-loop-helix (bHLH) transcription factor, is a strong promoter of metastatic spread and its expression is elevated in many advanced human carcinomas. However, the molecular events triggered by TWIST1 to motivate dissemination of cancer cells are largely unknown.

**Results:**

Here we show that TWIST1 induces the production of interleukin 8 (IL8), which activates matrix metalloproteinases and promotes invasion of breast epithelial and cancer cells. In this novel mechanism, TWIST1-mediated IL8 transcription is induced through the TWIST1 carboxy-terminal WR (Trp-Arg) domain instead of the classic DNA binding bHLH domain. Co-immunoprecipitation analyses revealed that the WR domain mediates the formation of a protein complex comprised of TWIST1 and the nuclear factor-kappaB (NF-κB) subunit RELA (p65/NF-κB3), which synergistically activates the transcriptional activity of NF-κB. This activation leads to increased DNA binding affinity of RELA to the IL8 promoter and thus induces the expression of the cytokine. Blockage of IL8 signaling by IL8 neutralizing antibodies or receptor inhibition reduced the invasiveness of both breast epithelial and cancer cells, indicating that TWIST1 induces autonomous cell invasion by establishing an IL8 antocrine loop.

**Conclusions:**

Our data demonstrate that the TWIST1 WR domain plays a critical role in TWIST1-induced IL8 expression through interactions with and activation of NF-κB. The produced IL8 signals through an autocrine loop and promotes extracellular matrix degradation to enable cell invasion across the basement membrane.

## Background

While treatment of the primary breast tumor is often well managed with surgery and radiation, metastatic spread to the brain, bones, liver and lungs frequently places women in an incurable state of disease [1]. The basic helix-loop-helix (bHLH) transcription factor TWIST1 was previously demonstrated to be a potent promoter of cancer cell dissemination into circulation and metastasis [2-7], providing an ideal target for investigation and a promising therapeutic target for intervention.

Based on its role in mesodermal development during mammalian embryogenesis [8,9], TWIST1 is proposed to induce an embryonic event termed epithelial-mesenchymal transition (EMT) in tumor cells to promote the expression of mesenchymal junction proteins in epithelial cells and reduce intercellular junctions in the meantime [2,10,11]. Induction of EMT enables epithelial cells to acquire the properties of mesenchymal lineages, including enhanced mobility and invasiveness that are tightly correlated with cancer metastasis [12,13]. Additionally, TWIST1 was shown to regulate the expression of AKT2 [14] and miRNA-10b [15], which are subsequently involved in the migratory and invasive properties of TWIST1-overexpressing cells. Eckert *et al. *recently reported that TWIST1 up-regulates the expression of platelet derived growth factor receptor, which in turn promotes the formation of invadopodia and matrix degradation [16], presenting the first evidence that TWIST1 causes extracellular matrix (ECM) remodeling. However, the mechanisms by which TWIST1 actively promote cell invasion are still largely unstudied.

TWIST1 is a class II member of the bHLH super family [17]. It homo- or heterodimerizes with class I HLH family members, such as E proteins (E12/E47), through the HLH domain, and binds DNA that contains the E-box sequence (CANNTG) via the basic domain to regulate gene expression [18]. Loss of function mutations in the bHLH domain in one allele of the *TWIST1 *gene cause Saethre-Chotzen Syndrome (SCS) in humans, an autosomal dominant craniofacial disease caused by gene haploinsufficiency [18-20], denoting the functional importance of the TWIST1 bHLH domain. TWIST1 also contains a highly conserved carboxy-terminus (C-terminus), the WR (Trp-Arg) domain (comprising the last 20 amino acid residues of TWIST1), which shares 100% sequence homology among jellyfish, *Xenopus*, mice and humans [17]. This region was shown to mediate the association between TWIST1 and Runx2, a zinc-finger protein, and prevent Runx2 from inducing premature osteoblast differentiation during bone development in mice [21]. However, whether the highly conserved WR domain plays any roles in TWIST1-induced cancer metastasis is an open question.

Here we describe a unique property of the TWIST1 WR domain in mediating IL8 production and breast cancer cell invasion. Using Gene Set Enrichment Analysis (GSEA) of genetic profiles and cytokine array analyses, we found that IL8 was specifically up-regulated by TWIST1 over-expression in the human breast epithelial cell line MCF10A as well as other breast cancer cell lines. We also discovered that TWIST1 activates the IL8 promoter, but, surprisingly, in a manner that is independent of its canonical DNA binding bHLH domain. In contrast, TWIST1-induced IL8 promoter activation is dependent on the C-terminal WR domain, through which TWIST1 interacts with the NF-κB subunit RELA. This physical association of TWIST1 and RELA activated and synergized the transcriptional activity of NF-κB and increased the binding affinity of RELA to DNA, which in turn stimulated IL8 expression. Finally, we demonstrate that the IL8 autocrine pathway in breast epithelial and cancer cells increases matrix metalloproteinase (MMP) production and enables autonomous invasion of the cells.

## Results

### TWIST1 induces IL8 cytokine production

To elucidate the molecular function of TWIST1 in human breast epithelial cells, we compared the gene expression profiles of a TWIST1 over-expressing mammary epithelial cell line (MCF10ATw) and the parental line from which it was derived (MCF10A). We analyzed these data by GSEA (2.07) using the C2 canonical pathway gene sets [22] on paired independent experiments performed in triplicate using these cell lines. The returned gene set permutation test showed a significant difference in the BioCarta_Cytokine_Pathway between MCF10A and MCF10ATw cells at a false discovery rate of 0.012 (Additional file 1, Figure S1A and D). The gene set included the pro-inflammatory cytokine interleukin 1 alpha (IL1α) and the leukocyte chemokine IL8 as two significant contributors to the BioCarta_Cytokine_Pathway enrichment, with respective ranks of 3 and 11 as determined by signal-to-noise metric among all genes on the gene array, and in concordance with TWIST1 expression (Additional file 1, Figure S1B, D).

**Figure 1:**
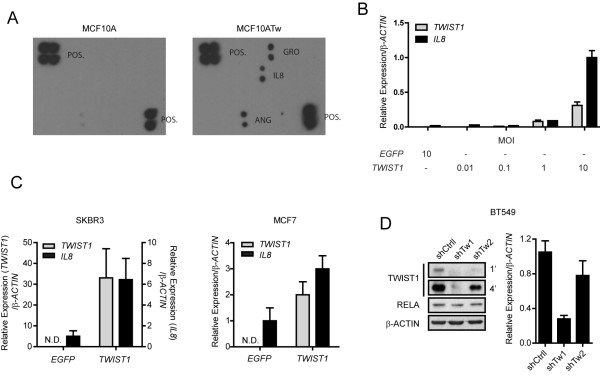
**TWIST1 induces IL8 Production**. (**A**) Cytokine array blotted with conditioned media collected from MCF10A or MCF10ATw cell cultures. Cytokines detected in MCF10ATw conditioned medium are shaded in Additional File 2, Table S1. POS, positive controls. (**B**) Relative mRNA levels of *TWIST1 *and *IL8 *normalized to β-ACTIN See Methods) in MCF10A cells 48 h post-transduction with adenoviruses that express TWIST1 (*Ad.TWIST*) at a serial multiplicity of infection (MOI). Cells transduced with *Ad.EGFP *(enhanced green fluorescent protein) at an MOI of 10 were used as controls. (**C**) Relative mRNA levels of *TWIST1 *and *IL8 *normalized to β-ACTIN as in (B) in SKBR3 and MCF7 cells 48 h post-transduction with *Ad.TWIST *as compared to *Ad.EGFP *(control). MOI = 5. (**D**) Relative mRNA levels of *IL8*, normalized as in (B), in BT549 cells with TWIST1 KD (right). Cells were transduced with lentiviruses that expressed a non-targeting shRNA (shCtrl) or shRNAs that targeted *TWIST1 *(shTw1, shTw2). Western blot (left) shows the levels of TWIST1 under these conditions. One and four minute exposures of the blot are shown. RELA is shown as a KD control. The data are from single representative quantification or images of at least three independent experiments.

To verify the up-regulation of these genes, we performed a cytokine array blot with conditioned media collected from MCF10A or MCF10ATw cell cultures. In this experiment, IL8, along with growth related oncogene (GRO) and angiogenin (ANG), showed the most pronounced up-regulation in MCF10ATw cells; however, secreted IL1α was not detected (Figure 1A, Additional file 2, Table S1). Quantification of IL8 revealed 34-fold greater mRNA expression and 3-fold greater secretion of IL8 protein into culture media of MCF10ATw relative to MCF10A cells (Additional file 1, Figure S1C). To test if IL8 was selectively up-regulated in the MCF10ATw stable cell line, we transiently transduced MCF10A parental cells with adenoviruses that expressed TWIST1 at serial dilutions and observed that IL8 transcripts were up-regulated in a dose dependent manner that correlated with the levels of TWIST1 expressed (Figure 1B). Since IL8 was up-regulated at both the mRNA and protein levels, indicating that IL8 is a downstream target of TWIST1, we, therefore, focused on elucidating the regulatory pathway of IL8.

To evaluate the induction of IL8 by TWIST1 under pathological conditions, we expressed TWIST1 in the SKBR3 (epidermal growth factor receptor 2 positive, ERBB2/HER2+) and MCF7 (estrogen receptor positive, ER+) cell lines, which represent two different subtypes of breast cancer, and observed respective 20-fold and 4-fold increases in IL8 transcript levels (Figure 1C). The difference in IL8 transcript levels between these two cell lines may be due to the influence of ERBB2 expression on NF-κB transcriptional activity [23]. In addition, short hairpin RNA (shRNA)-mediated knock-down (KD) of endogenous TWIST1 reduced the expression of IL8 mRNA in BT549 cells (Figure 1D), a triple-negative breast cancer cell line (progesterone receptor negative (PR-), ER- and HER2-) that expresses high endogenous levels of both TWIST1 and IL8. This reduction in IL8 expression correlated with the amount of TWIST1 protein knocked down (Figure 1D). Taken together, these data show that TWIST1 regulates and maintains IL8 expression in breast epithelial and cancer cells.

### TWIST1-induced IL8 transcription is mediated by the TWIST1 WR domain

**Figure 2:**
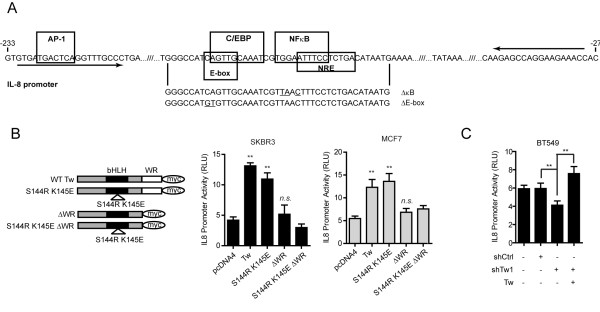
**TWIST1 induces IL8 promoter activity through the WR domain**. (**A**) Illustration of the IL8 promoter proximity region. Boxed sequences are consensus binding sites of indicated transcription factors. Mutations (underlined) introduced into the NF-κB and TWIST1 binding sites (E-Box) to generate the ΔκB and ΔE-box promoter constructs (See Additional File 3, Figure S2A) are shown. Arrows indicate the positions of primers used for amplification of the IL8 promoter for ChIP assays (Figure5B-D). (**B**) Relative activities of IL8 promoter-driven luciferase in SKBR3 (middle) and MCF7 (right) cells. Cell lysates were collected 24 h post-transfection with TWIST1 wild-type (WT Tw) or S144R K145E, ΔWR or S144R K145E ΔWR mutant TWIST1 constructs (illustrated on the left) and dual luciferase assays were performed. n.s., not significant. (**C**) Relative luciferase activities of BT549 cells stably transduced with shCtrl or shTw for TWIST1 KD, with or without the expression of exogenous TWIST1. The data are from single representative experiments. Mean ± SD, n = 3, ** *P *≤ 0.01.

To investigate whether TWIST1 activates IL8 transcription, we cloned the IL8 minimal promoter [24], which contains a putative TWIST1 binding site (an E-box sequence, CAGTTG) and a consensus κB response element, into a luciferase reporter construct (Figure 2A). SKBR3 and MCF7 cells transfected with this IL8 promoter construct displayed a consistent increase in luciferase activities when co-transfected with TWIST1, indicating transcriptional activation of IL8 (Figure 2B). Correspondingly, in BT549 cells, TWIST1 KD resulted in a decrease in IL8 promoter activity, and this activity was rescued upon exogenous TWIST1 expression (Figure 2C).

To further clarify whether TWIST1 directly binds and activates the IL8 promoter, we transfected SKBR3 and MCF7 cells with an IL8 promoter construct that was mutated at the putative TWIST1 binding E-box (Figure 2A, ΔE-box, CA:GT). In this experiment, TWIST1 failed to stimulate luciferase transcription (Additional file 3, Figure S3A), indicating that integrity of the E-box sequence is required for IL8 promoter activation. However, we were concerned that these mutations may affect the binding of C/EBP, a known regulator of IL8 because the E-box sequence heavily overlaps with the C/EBP binding site [25]. Therefore, we generated two DNA binding deficient TWIST1 mutants by introducing the S144R/K145E and R118C mutations (Figure 2B and S2B). These naturally occurring mutations found in SCS patients abolish the DNA binding ability of TWIST1/E12 heterodimers without affecting its subcellular localization [18]. Surprisingly, both the S144R/K145E (Figure 2B) and R118C (Additional file 3, Figure S2B) TWIST1 mutants stimulated the IL8 promoter at similar levels as wild type (WT) TWIST1 in SKBR3 and MCF7 cells (Figure 2B and Additional file 3, Figure S2B). To further validate that the DNA binding ability of TWIST1 is not required for IL8 promoter activation, we completely removed the bHLH domain of TWIST1 (ΔbHLH) and still observed IL8 promoter activity induced by this mutant comparable to that of wild type TWIST1 in MCF7 cells (Additional file 3, Figure S2B). More importantly, when we performed chromatin immunoprecipitation (ChIP) assays with solubilized chromatin collected from MCF10A cells transfected with S144R/K145E DNA-binding deficient TWIST1 mutant, we did not detect any statistically significant enrichment of mutant TWIST1 capable of activating IL8 expression on the IL8 promoter (See later results, demonstrating that the activation of IL8 promoter is independent of DNA binding activity of TWIST1.

To better understand which region of the TWIST1 protein is responsible for IL8 promoter activation, we decided to remove the last 20 amino acids at the C-terminal end of TWIST1, which comprise its highly conserved WR domain (ΔWR), and transfected this ΔWR mutant protein into SKBR3 and MCF7 cells. Intriguingly, unlike the DNA binding deficient TWIST1 mutants, the ΔWR truncation mutant protein completely lost its ability to activate the IL8 promoter. Furthermore, combining the S144R/K145E mutations with the WR deletion showed similar activation of the IL8 promoter as compared to ΔWR alone (Figure 2B), illustrating that the WR domain is essential for TWIST1-induced IL8 expression.

### TWIST1-induced IL8 expression is mediated by RELA

**Figure 3:**
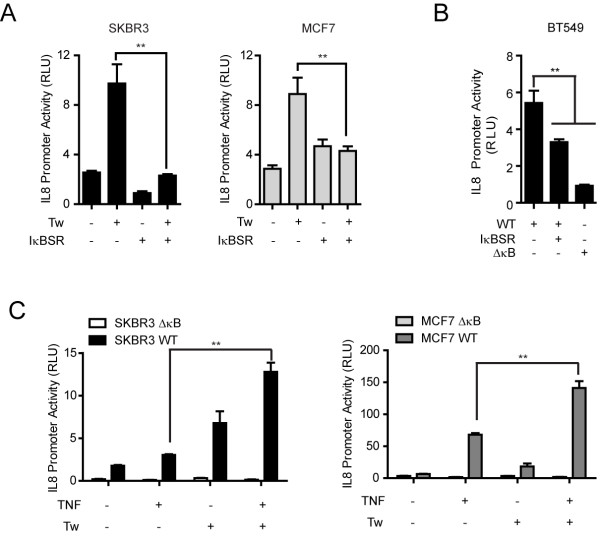
**NF-κB mediates TWIST1- induced IL8 promoter activation**. (**A**) IL8 promoter-driven relative luciferase activities of SKBR3 (left) and MCF7 (right) cells 24 h after transfection with TWIST1 (Tw) and/or IκBSR. (**B**) Relative luciferase activities of BT549 cells 24 h post-transfection with the IL8 WT promoter with or without IκBSR, or ΔκB IL8 mutant promoter alone. (**C**) Relative luciferase activities of SKBR3 (left) and MCF7 (right) cells transfected with ΔκB or WT IL8 promoter in the presence or absence of TWIST1 and/or TNF stimulation (40 ng/ml). Data are from single representative experiments. Mean ± SD, n = 3, ** *P *≤ 0.01.

It was reported by Sosic *et al. *that TWIST1 and 2 regulate the transcriptional activity of NF-κB in mice and specifically TWIST2 does so through its C-terminal region, which contains a WR domain [26]. Given that NF-κB is a central regulator of IL8 [24] and that the WR domain is highly conserved between TWIST1 and TWIST2, we hypothesized that NF-κB is involved in TWIST1-mediated IL8 promoter activation. To address this hypothesis, we made use of a dominant negative (DN) form of IκBα (S32A S36A super repressor, IκBSR) that constitutively suppresses the activation of NF-κB [27], and found that TWIST1 could no longer activate the IL8 promoter when co-transfected with this suppressor in both SKBR3 and MCF7 cells (Figure 3A). To determine if this was due to the inference of IκBSR on the expression or activity of TWIST1, we examined the expression of TWIST1 in the nuclei of cells co-transfected with IκBSR, and in fact found greater amounts of TWIST1 protein in the nuclear fractions of these cells than cells transfected with TWIST1 alone (Additional file 3, Figure S2C). Additionally, in BT549 cells, which express high endogenous levels of TWIST1, transfection of IκBSR showed attenuated IL8 promoter activity, which was completely abolished by mutations in the κB consensus site that disrupts binding of NF-κB to the IL8 promoter [25] (Figure 2A underlined ΔκB, 3B), indicating that NF-κB transcriptional activity is crucial in the expression regulation of IL8 in these cells.

To compare the role of TWIST1 and tumor necrosis factor alpha (TNF-α), an activator of NF-κB, on IL8 promoter activation, we treated SKBR3 and MCF7 cells transfected with the IL8 promoter construct and DNA vector or TWIST1 with TNF-α. Although the IL8 promoter was activated to greater levels by TNF-α alone than by TWIST1 transfection in both cell lines, the expression of TWIST1 synergized the effect of TNF-α on IL8 promoter activation in both cell lines. Moreover, mutations in the κB consensus binding site eliminated this synergy, indicating that the synergistic effect of TWIST1 and NF-κB is dependent upon NF-κB/DNA binding (Figure 3C). The above data also suggest that TWIST1 activates the transcriptional activity of NF-κB, possibly through a mechanism upstream of IκBα, different from the canonical TNF-α pathway.

**Figure 4:**
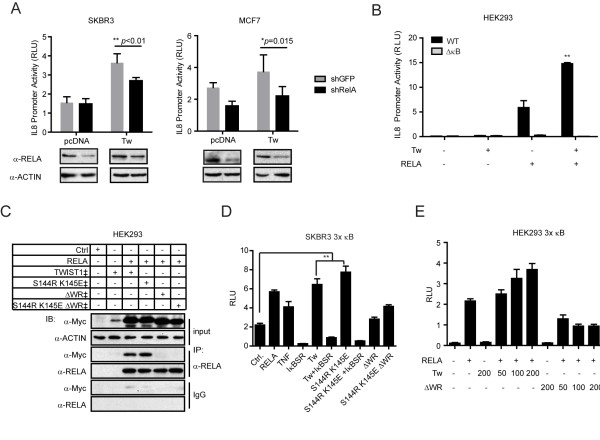
**TWIST1 interacts with RELA and induces NF-κB transcriptional activity through the TWIST1 WR domain**. (**A**) IL8 promoter-driven relative luciferase activities of SKBR3 and MCF7 RELA KD cells transfected with or without TWIST1. Cells were transfected with control shRNA (shGFP) or shRNA against *RELA *(shRelA) 24 h before the transfection of IL8 promoter construct with TWIST1 or pcDNA (vector) control followed by luciferase activity measurement 24 h later. Western blots (below) show the representative KD levels of RELA in these cell lines. (**B**) Relative luciferase activities in HEK293 cells 24 h post-transfection with TWIST1 and/or RELA. WT, IL8 WT promoter; ΔκB, ΔκB IL8 mutant promoter (shown in Figure 2A). (**C**) Co-IP experiments with α-RELA antibodies or IgG control. Antibodies were incubated with lysates collected from HEK293 cells 24 h post-transfection with RELA and WT or mutant TWIST1. Cells that were not transfected or transfected with WT TWIST1 only were used as negative controls. ‡, presence of Myc-tag on the TWIST1 proteins. (**D**) Relative luciferase activities driven by 3x NF-κB response elements in SKBR3 cells 24 h post-transfection with indicated constructs or treated with TNF (40 ng/ml). (**E**) Relative activities of luciferase driven by 3x NF-κB response elements in HEK293 cells 24 h post-transfection with RELA (50 ng) and WT or ΔWR TWIST1. The amounts of TWIST1 DNA constructs transfected are indicated below (ng). Total amount of DNA transfected was kept constant with sham vectors. (A), (B), (D) and (**F**) are from single representative experiments. Mean ± SD, n = 3, *P ≤ 0.05, ** *P *≤ 0.01.

To further understand the mechanism by which the activation and synergy of NF-κB occurs, we focused on the NF-κB subunit RELA, which is a conserved binding partner of TWIST1 [26,28]. To address whether RELA is the NF-κB subunit that mediates TWIST1-induced IL8 expression, we performed shRNA-mediated KD of RELA in SKBR3 and MCF7 cells, and found that IL8 promoter-driven luciferase activity under TWIST1 over-expression was attenuated by RELA KD in both cell lines (Figure 4A). When we performed double KD of TWIST1 and RELA in BT549 cells, we observed a slight decrease of the IL8 promoter activity compared to TWIST1 KD (Additional file 3, Figure S2D), indicating that RELA alone is partially responsible for the basal expression of IL8. We also noticed that the KD of RELA in TWIST1 over-expressing cells was less efficient than in TWIST1-null or low expressing cells, suggesting that TWIST1 expression may stabilize RELA protein when its expression is reduced without affecting its overall basal expression levels (Figure 4A and Additional file 3, Figure S2E). Additionally, forced expression of TWIST1 in HEK293 cells, which do not express detectable levels of TWIST1 or RELA (Figure 4C and Additional file 3, Figure S2F), failed to stimulate IL8 promoter activity; whereas transfection of these cells with RELA activated the IL8 promoter, which was further synergized by the co-transfection with TWIST1 (Figure 4B). Furthermore, mutations in the κB consensus binding site abolished this synergistic effect between TWIST1 and RELA (Figure 4B), indicating that the association of NF-κB with DNA is necessary for IL8 transcriptional activation and synergism induction by TWIST1.

The TWIST1 WR domain was previously shown to mediate the interaction between TWIST1 and non-bHLH protein partners, such as Runx2 [21]. An association between recombinant murine TWIST1 and RELA was also previously reported [26]. Therefore, we speculated that the TWIST1 WR domain bridges an interaction between human TWIST1 and RELA. To address this, we performed RELA co-immunoprecipitation (co-IP) experiments using HEK293 cells transfected with WT or mutant TWIST1 using α-RELA antibodies (Figure 4C). Consistent with earlier reports, WT human TWIST1 co-immunoprecipitated with RELA; and the TWIST1 bHLH mutant (S144R K145E) was also precipitated by α-RELA antibodies in HEK293 cells transfected with the TWIST1 mutant and RELA. However, neither the ΔWR nor S144R K145E ΔWR TWIST1 mutant could be detected by α-RELA antibodies in co-IP experiments when equal amounts of RELA were precipitated. Collectively, these results demonstrate that the TWIST1 WR domain is necessary for the association between RELA and TWIST1, and is crucial for transcriptional activation of TWIST1-induced IL8 expression.

To determine if this interaction between RELA and TWIST1 regulates the activity of NF-κB, we used a luciferase construct driven by an artificial promoter that contains three repeats of the κB consensus sites (3x κB). In this experiment, both WT and S144R K145E TWIST1 stimulated the κB consensus site in SKBR3 cells, but this stimulation was completely blocked by IκBSR expression, indicating that the activation was mediated by the endogenous NF-κB (Figure 4D). In contrast, neither the ΔWR nor S144R K145E ΔWR TWIST1 mutant could stimulate NF-κB transcriptional activity in a similar manner in luciferase assays (Figure 4D). Furthermore, the increase in NF-κB transcriptional activity was dependent upon the amount of TWIST1 being expressed (Figure 4E), whereas ΔWR TWIST1 in any given amount failed to activate NF-κB transcriptional activity in HEK293 cells transfected with the 3x κB luciferase construct (Figure 4E). These results demonstrate that the TWIST1 WR domain, which mediates the association between TWIST1 and RELA, is essential for the activation of NF-κB transcriptional activity and synergy.

### TWIST1 increases the association of RELA with the IL8 promoter

**Figure 5:**
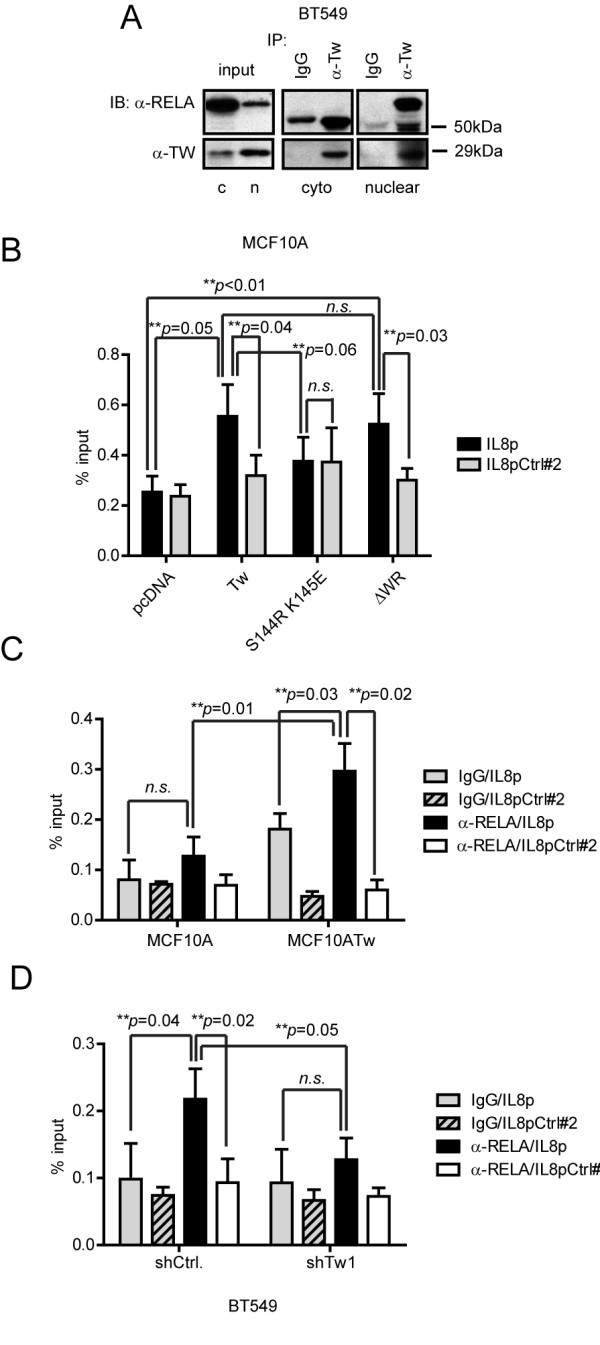
**TWIST1 enhances the association of RELA with the IL8 promoter**. (**A**) Fractionation-coupled Co-IP experiments (upper panel) with BT549 cell lysates using α-TWIST1 antibodies or IgG control. Cyto or c, cytoplasmic; n, nuclear. (**B**) ChIP assays using α-TWIST1 antibodies with solublized chromatin collected from MCF10A cells transfected with pcDNA, wild type TWIST1, S144R K145E or DWR TWIST1 mutant for 48 h. (**C**) ChIP assays with MCF10A and MCF10ATw cell lysates (left), or lysates from BT549 cells transduced with shCtrl or shTw (right), using α-RELA antibodies and IgG control. (**D**) ChIP assays with BT549 cell lysates using α-TWIST1 (left) or α-RELA antibodies and IgG controls. Mean ± SD, n = 3. IL8p, IL8 promoter amplified with primers flanking the E-box and kB binding sites (Figure 2A); IL8pCtrl#2, IL8 promoter enhancer region amplified with primers 2 kb upstream of transcription initiation site (primer sequences see Methods).

To confirm the physiologic relevance of the association between TWIST1 and RELA, we performed co-IP experiments with BT549 cells, which express endogenous TWIST1. Using α-TWIST1 antibodies, we found that RELA was exclusively pulled down with TWIST1 in the nuclear but not the cytosolic fraction of BT549 cells (Figure 5A), suggesting that TWIST1 and RELA form a protein complex in the nuclear compartment.

Next, to understand if the TWIST1/RELA protein complex is associated with the IL8 promoter sequence and whether this protein/DNA association is essential for promoter activation, we performed ChIP assays using α-TWIST1 antibodies with MCF10A cells transfected with WT TWIST1, S144R/K145E or ΔWR TWIST1 mutants (Figure 5B). We found that although the WT and the ΔWR TWIST1 mutant were associated with the IL8 promoter, the S144R/K145E DNA binding mutant is not presented at the same region, confirming that these point mutations indeed disrupt the DNA binding ability of the protein. More importantly, it demonstrates that the activation of RELA by TWIST1 can be achieved away from the IL8 promoter DNA and that the direct or indirect association of TWIST1 via RELA to the IL8 promoter is not required for IL8 transcription activation. In addition, we also observed that there is no difference in the enrichment of the IL8 promoter between WT TWIST1 and ΔWR-expressing cells indicating that the interaction between TWIST1 and RELA does not appear to affect the ability of TWIST1 to become associated with the IL8 promoter, presumably through the E-box sequence (Figure 5B, Additional file 3, Figure S2G).

We further compared the association of RELA with the IL8 promoter between MCF10A and MCF10ATw cells and detected statistically enriched signals of RELA on the IL8 promoter in MCF10ATw relative to MCF10A cells (Figure 5C), which indicates that the binding affinity of RELA to the IL8 promoter is greatly enhanced in the presence of TWIST1. Reciprocally, the percentage of RELA-associated IL8 promoter was significantly reduced in BT549 cells when TWIST1 was KD (BT549.shTw1) compared to those transduced with non-targeting shRNA control (BT549.shCtrl) (Figure 5E, right). Together, these data demonstrate that TWIST1 increases the recruitment of RELA to the IL8 promoter.

### Elevated levels of IL8 enhance MMP activation and increase cellular invasive potential of breast epithelial cells

**Figure 6:**
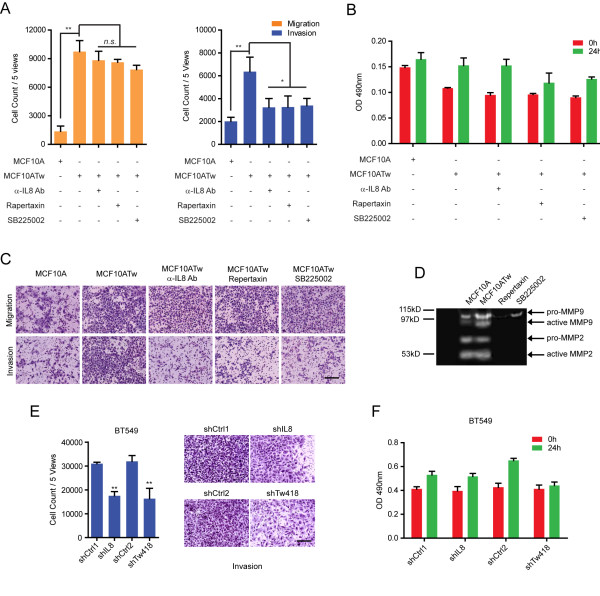
**IL8 increases cellular invasive potentials**. (**A**) Transwell migration (left) and invasion (right) assays of MCF10A and MCF10ATw cells towards 20% horse serum for 24 h. MCF10ATw cells were treated as indicated. α-IL8 Ab, IL8 neutralizing antibody, 10 μg/ml; SB225002, 10 nM; Repertaxin, 400 μM; n.s. not significant. Mean±SD, n = 3, *P ≤ 0.1, **P ≤ 0.05. (**B**) Proliferation assays for MCF10A, MCF10ATw and MCF10ATw cells cultured as indicated with conditions described in (A) for 24 h. Mean±SD, n = 3. (**C**) Representative images of cells from transwell migration and invasion experiments described in (A). Scale bar, 160 μm. (**D**) Gelatin zymography assays of conditioned media collected from MCF10A, MCF10ATw or MCF10ATw cell cultures treated with indicated compounds for 24 h. SB225002, 100 nM; Repertaxin, 400 nM. (**E**) Transwell invasion assays (left) of BT549 cells towards 20% fetal bovine serum for 24 h. Cells were stably transduced with control shRNAs (shCtrl1, shCtrl2), shRNA against IL8 (shIL8) or TWIST1 (shTw). Mean ± SD, n = 3, **P ≤ 0.05. Representative images (right) of BT549 cells that invaded through Matrigel. Scale bar, 160 μm. (**F**) Proliferation assays (24 h) for BT549 cells stably transduced with shCtrls, or shRNA against IL8 (shIL8) or TWIST1 (shTw) under conditions described in (E). Mean±SD, n = 3.

Under physiological conditions, IL8 activates endothelial cells and neutrophils to produce and release MMPs to enhance their migration and invasion in order to facilitate angiogenesis and extravasation, respectively [29,30]. These observations suggest that IL8 produced by TWIST1-expressing breast cells may also alter their migratory and invasive potentials, features seen with many invasive cancer cells. To examine this possibility, we compared MCF10A and MCF10ATw cells and found that expression of TWIST1 significantly increased the migratory and invasive potential of MCF10A cells in transwell assays towards serum-rich media (Figure 6A, C). When MCF10ATw cells were treated with IL8 neutralizing antibodies or inhibitors against the IL8 receptors (SB225002, a CXCR2 receptor inhibitor [31], Repertaxin, a CXCR1 and CXCR2 inhibitor [32]), the number of invasive cells was reduced by 50%. Interestingly, no significant difference was seen in the number of migratory cells in response to IL8 signaling blockage (Figure 6A, C), indicating that TWIST1 mediates the production of IL8 to induce ECM degradation but not to enhance the cell's motility. To test whether blockage of the IL8 autocrine pathway affected cell division in the migration/invasion assays, we performed cell proliferation assays and found cell growth was not affected by the treatment of neutralizing antibodies or inhibitors (Figure 6B), verifying that the decrease in number of invaded cells was not a result of reduced proliferation.

Because IL8 autocrine signaling significantly altered the invasive properties of MCF10ATw cells, we analyzed the expression and activation levels of MMPs, which play important roles in ECM degradation. Using gelatin zymography, we found that the production and activation of MMP9 were greatly enhanced by TWIST1 over-expression in MCF10A cells. Addition of Repertaxin or SB2255002 to MCF10ATw cell cultures markedly reduced the expression and activation of both MMP2 and MMP9 to below detectable levels (Figure 6D). These results indicate that TWIST1 over-expression establishes an IL8 autocrine loop that regulates the production and activation of MMP2 and 9.

To investigate whether IL8 also mediates cellular invasiveness in cancer cells, we performed shRNA-mediated KD of IL8 in BT549 cells, and found a 40% reduction in the number of invasive cells as compared to the non-targeting shRNA control. This reduction in the number of invasive cells as a result of IL8 KD was similar to that of the TWIST1 KD of BT549 cells in comparison to both non-targeting shRNA controls (Figure 6E). In addition, neither IL8 nor TWIST1 KD of BT549 cells displayed significant differences in their proliferation rates during the course of the invasion assay (Figure 6F). Collectively, these data demonstrate that TWIST1-induced IL8 production regulates the invasive properties of breast epithelial and cancer cells through an autocrine loop.

### *TWIST1 *and *IL8 *are selectively co-expressed in the basal subtype of human breast cancers

**Figure 7:**
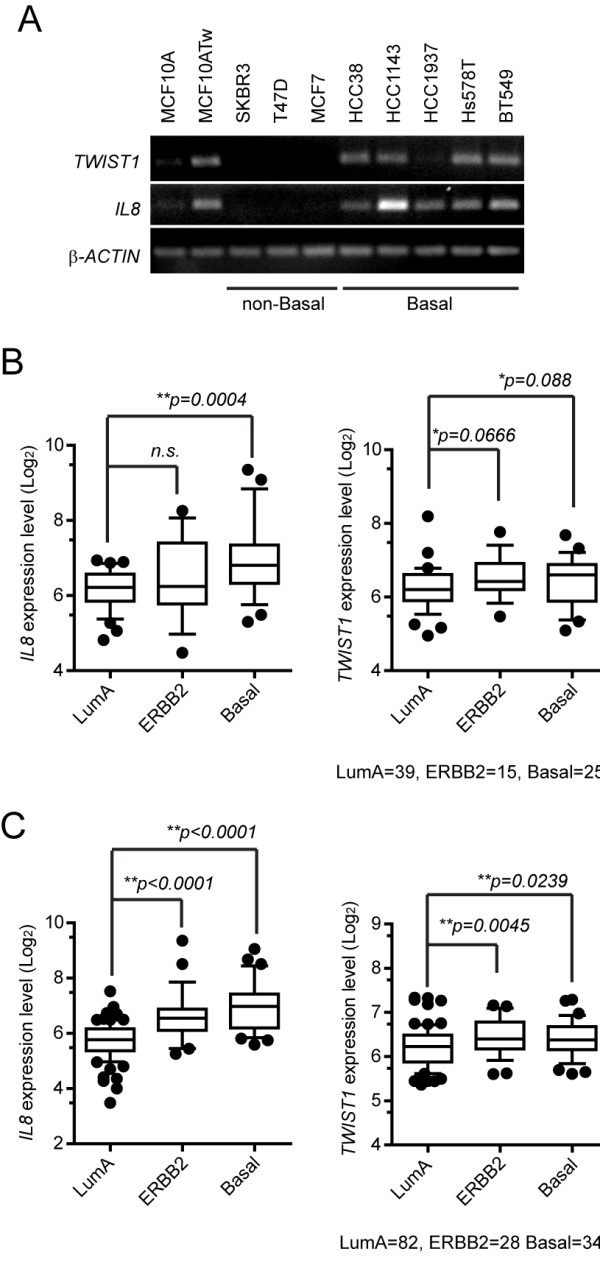
***TWIST1 *and *IL8 *expression is enriched in the basal subtype of human breast cancers**. (**A**) Reverse-transcription PCR of indicated cell lines for *TWIST1 *and *IL8 *with β-ACTIN as control. (**B**) Relative mRNA levels of *IL8 *and *TWIST1 *of luminal A (LumA), ERBB2/HER2+ (ERBB2) and basal subtypes from STOCKHOLM human breast tumor dataset (total n = 159). Boxes represent the lower quartile, median and upper quartile; Whiskers represent the tenth and ninetieth percentiles; and dots represent outliers. *P*-values were calculated using Mann-Whitney U test. (**C**) Relative mRNA levels of *IL8 *and *TWIST1 *of LumA, ERBB2 and basal subtypes from UPPSALA human breast tumor dataset (total n = 253). Graphs were plotted and *P*-values calculated as described in (B).

We compared the expression of *TWIST1 *and *IL8 *in multiple breast cancer cell lines, which revealed that both *TWIST1 *and *IL8 *are expressed in the basal but not in the non-basal breast cancer cell lines (Figure 7A). Although the amounts of *IL8 *transcript did not appear to be proportional to the detected levels of *TWIST1*, which is likely due to the presence of other IL8 regulators in these cell lines, these results indicate an overall correlation between *TWIST1 *and *IL8 *expression in the aggressive basal subtype of human breast cancers.

To determine the relevance of the *in vitro *mechanistic studies in relation to patient samples, we analyzed the relative expression levels of *TWIST1 *and *IL8 *in the STOCKHOLM (GSE1456) and UPPSALA (GSE3494) human breast tumor datasets (with a total of 412 samples from both datasets combined). These samples were divided into luminal A, luminal B, normal-like, ERBB2 and basal subtypes based on their molecular intrinsic signatures identified by Calza *et al. *([33], personal communication with Dr. Pawitan). Results from these analyses demonstrate significantly higher levels of *IL8 *expression in the basal compared to the luminal A subtype in both datasets, which was also seen in the ERBB2 subtype in the UPPSALA dataset (Figure 7B, C). Moreover, although the overall differences in relative expression levels of *TWIST1 *were small across different subtypes, *TWIST1 *expression was significantly elevated in the basal as well as the ERBB2 subtypes in both datasets when compared to luminal A subtype (Figure 7B, C). More importantly, the co-expression of *TWIST1 *and *IL8 *is correlated in the basal and ERBB2 subtypes, the two most aggressive subtypes of human breast cancers [33], supporting the important roles of the transcription factor and the cytokine in the pathology of advanced breast cancers.

## Discussion

Herein, we show that TWIST1 up-regulates IL8 expression to induce cell autonomous invasion through the conserved C-terminal WR domain. The WR domain mediates the association of TWIST1 and RELA, which is essential for TWIST1-induced stimulation and synergism of NF-κB transcriptional activity and IL8 production. Additionally, TWIST1 forms a protein complex with RELA and enhances the association of RELA with the IL8 promoter, thus inducing IL8 expression. Finally, TWIST1-mediated secretion of IL8 establishes an autocrine loop in breast cells to regulate MMP production and cell invasion.

TWIST1 is commonly characterized by its bHLH domain, which is thought to be responsible for the transcriptionally regulated events controlled by this molecule [2,10,14-16]. However, the functional domains of TWIST1 have not been thoroughly studied and many questions are unanswered regarding their potential interacting partners, which in turn can be highly valuable for understanding the mechanism of cancer cell dissemination. Here we demonstrate that instead of direct IL8 gene activation, TWIST1 interacts with RELA, a non-HLH binding partner, and activates/synergizes transcriptional activity of NF-κB to up-regulate the NF-κB downstream gene target, IL8, which in turn regulates MMP production and cell invasion. This example indicates that TWIST1 can recruit non-HLH transcription factors to form protein complexes and modify gene expression downstream of this partner, which is supported by previous developmental and biochemical studies showing that TWIST1 can regulate the activity of its interacting co-factors [21,34,35].

RELA is a subunit of the NF-κB complex, which is a central mediator of inflammatory responses [36] and causes many pathophysiological conditions upon activation [37], including tumorigenesis and metastasis [38,39]. Previous reports indicated that during normal mesodermal tissue development, TWIST1 and 2 inhibited the transcriptional activity of NF-κB and suppressed expression of the pro-inflammatory cytokines TNF-α and IL-1β [26]. In contrast, our results show that in breast tumor cell lines, TWIST1 stimulates NF-κB through the TWIST1 WR domain and up-regulates the expression of the NF-κB downstream target gene IL8. These findings are in agreement with an earlier report indicating that TWIST1 synergizes the transcriptional activity of NF-κB in a manner that is independent of its bHLH domain [28]. The seemingly contradicting data in normal mesodermal tissues and breast tumor cell lines may indicate there are additional players modifying the functional relationships between TWIST1 and RELA during different processes in development. We hypothesize that the potential regulatory mechanisms may involve the initial availability of additional co-factors in a given cell type as well as the transcription factor binding sites present on specific target promoters. As an example, during dorsal-ventral development in *Drosophila *embryos, the cell types along the lateral wall of the embryo are determined by the expression of genes [40,41], which are differentially regulated by gradients of the morphogens Dorsal (orthologue of RELA) and Twist, as well as the proximity of the binding sites on promoters of these genes [42]. Here, the selective induction of IL8 among many cytokines also argues for the importance of promoter organization as a critical factor that determines gene regulation by TWIST1 and NF-κB as in comparison to a non-cytokine NF-κB target gene, IκBα, whose promoter was not enriched with either TWIST1 or RELA in our ChIP assays (data not shown).

Our data that TWIST1 activates RELA to induce IL8 expression suggest that in breast epithelial and cancer cells, the expression of TWIST1 can translate an extracellular signal, such as hypoxia, and signal intracellularly to modify the activity of endogenous RELA and induce IL8 expression specifically when there is no apparent extracellular signal for NF-κB activation. Furthermore, that TWIST1 can synergize the IL8 promoter activation induced by TNF-α indicates that TWIST1- and TNF-α-induced RELA/NF-κB activation contain overlapping yet non-identical biological effects. Finally, Pham *et al. *[43] confirm Sosic *et al.*'s findings [26] that TWIST1 is a downstream target of RELA, yet they found that TWIST1 acts as a RELA effector protein to block programmed cell death mediated by TNF-α treatment, implying that the functional relationships between TWIST1 and TNF-α in NF-κB activation can depend on the genes being regulated and the physiological conditions of the specific cell type.

Despite some discrepancies [44], IL8 expression was elevated in both invasive cancer cells [45,46] and in the sera of patients with aggressive cancers [47,48]. It is well known that IL8 induces potent neutrophil chemotaxis [49], which causes cytoskeletal reorganization, as well as release and activation of MMPs from neutrophils to induce directed migration of the granulocytes [50]. Here we show that TWIST1-induced IL8 expression in breast epithelial cells up-regulates MMP production and enhances cellular invasive property without affecting migratory ability, suggesting that the biological effects of IL8 on motivating cytoskeleton remodeling are slightly different in cancer cells. This also implies that secreted IL8 primarily enhances the cellular ability to degrade basement membrane (laminin, the primary component of Matrigel), a phenomenon common in metastatic cancers. Blockage of IL8 signaling or knockdown of the cytokine with shRNAs against TWIST1 did not cause MCF10ATw (Figure 6C, top panel) or BT549 (Figure 6E, upper right) cells to lose their mesenchymal phenotype, indicating the reduced invasive properties resulted from IL8 signaling inhibition was not a result of reversion of EMT. This observation may be because of the fact that mesenchymal-epithelial transition is a lengthy process; however, immunoblots of epithelial and mesenchymal markers of BT549 cells stably transduced with shIL8 or shTw did not appear to be morphologically or biochemically different from the BT549.shCtrl cells (data not shown), suggesting the increased cellular invasiveness mediated by IL8 expression is independent of the EMT process.

The fact that IL8 is a chemokine for neutrophils is another important element for tumorigenesis and the development of tumor microenvironment. Neutrophils are short-lived multinuclear white blood cells that have a profound role in ECM degradation and are responsible for several diseases characterized by severe damage of tissue structure [51-53]. The expression of IL8 by tumor cells can cause infiltration of neutrophils (tumor-associated neutrophils, TAN), which has been evident for many years [54]. It was recently discovered that, like tumor-associated macrophages, TAN also displayed two phases of actions, switching from the anti-tumorigenic to the proto-tumorigenic stage [55], providing evidence of the involvement of neutrophils in cancer progression. Moreover, IL8 is a known effective pro-angiogenic factor [56] that promotes endothelial cell survival and MMP production critical for vascularization [30]. This characteristic of IL8 may partially be responsible for TWIST1-associated tumor angiogenesis that was previously noted by our group and collaborators in TWIST1 over-expressing MCF7 breast tumor cells [57]. Overall, increase of basal expression of IL8 can bring forth its pleiotropic effects in affecting tumor progression, remodeling of the tumor microenvironment and the formation of cancer metastasis.

## Conclusions

Our study demonstrates that the TWIST1 WR domain is functionally critical to TWIST1-induced cell invasion. This domain is essential for the interaction between TWIST1 and the NF-κB subunit RELA, which is key to IL8 induction. TWIST1-mediated up-regulation of IL8 leads to MMP production and ECM degradation, resulting in enhanced cell-autonomous invasion.

## Methods

### Cell culture and transfection

The MCF10ATw cell line was generated by stable transfection [58] of pcDNA3-TWIST [59] into MCF10A cells (an immortalized normal human breast epithelial cell line, American Type Culture Collection, ATCC, Manassas, VA, USA). MCF10ATw and MCF10A cells were cultured as previously described [60]. HEK293, SKBR3, MCF7 and BT549 cell lines were purchased from ATCC and cultured using ATCC's recommended protocols and authentication methods. All cells were maintained at 37°C, 5% CO_2_, 90% humidity in a tissue culture (TC) incubator. Transfections were performed using Lipofectamine 2000 (Invitrogen, Carlsbad, CA, USA) according to the manufacturer's instructions.

### Microarray analysis

Triplicate samples of MCF10A and MCF10ATw cells were used for expression profiling with the Affymetrix Human Genome U133 Plus 2.0 array, and data collection was performed in the Microarray Core Facility at City of Hope. RNA was extracted using TRIzol reagent (Invitrogen) and analyzed for integrity using an Agilent Bioanalyzer 2100 (Agilent Technologies, Wilmington, DE, USA). Double stranded cDNA was reverse transcribed using total RNA (5 μg), the GeneChip^® ^Expression 3'-Amplification Reagents One-Cycle cDNA Synthesis Kit (Affymetrix, Santa Clara, CA, USA) and oligo-dT primers containing a T7 RNA polymerase promoter. A 1:10 dilution of Poly-A controls (2 μl) was added as an internal control for the synthesis. Double-stranded cDNA was used as a template to generate biotinylated cRNA using the GeneChip^® ^Expression 3'-Amplification Reagents for *in vitro *transcription labeling (Affymetrix). Biotin-labeled cRNA was fragmented following the Affymetrix protocol. Hybridization cocktails contained 15 μg fragmented cRNA, 5 μl 3 nM control oligonucleotide B2, 15 μl 20X eukaryotic hybridization controls, BSA (0.5 mg/ml), herring sperm DNA (0.1 mg/ml), dimethyl sulfoxide (DMSO) (10%), and hybridization buffer for a final volume of 300 μl. Hybridization cocktails (200 μl) were hybridized (45°C, 16 h) to HG U133 Plus 2.0 Affymetrix arrays in an Affymetrix GeneChip Hybridization Oven 640. GeneChip arrays were washed with wash buffer (Affymetrix, santa Clara, CA, USA) and stained with streptavidin-phycoerythrin on an Affymetrix Fluidics Station 450, followed by scanning on an Affymetrix GeneArray 3000 scanner. Data were extracted using GeneChip Operating Software (version 1.4) (Affymetrix, Santa Clara, CA USA). Analysis of microarray data was performed using Partek Genomics Suite 6.4 (Partek, Inc., St. Louis, MO, USA) as follows. The Robust Multi-array Average (RMA) algorithm was adapted to normalize and summarize the intensities of probes into gene-level expression. A two-way ANOVA model was used with TWIST over-expression and scan dates as factors to identify the effect contributed mainly by TWIST. Only genes with *P*-value < 0.05 and |fold change| >2 were considered significantly differentially expressed. Genes were further analyzed using Gene Set enrichment Analysis (GSEA) to provide gene enrichment analysis and functional interpretation. A cytokine heat map was generated based on cytokine-related gene sets available from GSEA.

### ELISA

Conditioned media from cell cultures with equal numbers of seeded cells (8 × 10^5^, six-well plate) cultured for 24 h were collected for ELISA (eBiosciences, San Diego, CA, USA).

### Cytokine arrays, immunoblotting, and co-immunoprecipitations

Cytokine arrays (RayBiotech, Inc., Norcross, GA, USA) were blotted using the manufacturer's instructions. For immunoblotting, proteins were resolved by SDS-PAGE and probed with anti-E-Cad (Cell Signaling, Danvers, MA, USA), anti-N-Cad (Abcam, San Francisco, CA, USA), anti-Vimentin (R&D Systems, Minneapolis, MN, USA), anti-TWIST1 (Abcam), anti-RELA (Santa Cruz Biotechnology Inc., Santa Cruz, CA, USA), anti-Myc (EMD, San Diego, CA, USA) and anti-β-actin (Sigma-Aldrich, St. Louis, MO, USA) antibodies. For immunoprecipitation, HEK293 cells were solubilized in RIPA buffer, the lysate centrifuged (10,000 × *g*, 10 minutes) and the supernatant containing the soluble proteins collected. Protein lysates (100 μg) were first pre-cleared with normal IgG and protein A/G plus conjugated agarose beads (Santa Cruz Biotechnology, Inc.), then incubated with new beads and antibodies of interest overnight (4°C, on a rocker). Beads were washed five times (RIPA buffer) and boiled in Laemmli loading buffer in the presence of Dithiothreitol for further analysis. BT549 cells collected for fractionation-coupled co-IP were first resuspended in KCl (10 mM) hypotonic buffer and lysed with 0.4% IGEPAL (NP-40 substitute); nuclei were collected by centrifugation and solubilized with high salt buffer (0.4 M NaCl) by rocking (4°C, 1 h). Nuclear lysates were cleared by centrifugation (16,000 × *g*, 5 minutes) and the salt concentration was adjusted to 135 mM for co-IP experiments.

### RT-PCR and quantitative PCR

Total RNA was isolated with the RNeasy Mini Kit (Qiagen, Valencia, CA, USA). First strand cDNA was synthesized from 1 μg total RNA with the iScript cDNA synthesis kit (Bio-Rad, Hercules, CA, USA). PCR was performed for 35 cycles (95°C, 30 s; 57°C, 30 s; 72°C, 15 s) with *Taq *DNA polymerase (Invitrogen). Relative mRNA levels were quantified using SYBR supermix (Bio-Rad, Hercules, CA, USA) on an iCycler iQ5 for 40 cycles (95°C, 30 s; 57°C, 15 s; 72°C, 15 s) followed by default melting curve cycles and analyzed using IQ5 software by PCR baseline subtraction (Bio-Rad). The following primers were used: *TWIST1 *forward 5'-AGCAAGATTCAGACCCTCAAGC-3', reverse 5'-CTCCATCCTCCAGACCGAGA-3'; *IL8 *forward 5'-CTGTCTGGACCCCAAGGAAAACT-3', reverse 5'-GCAACCCTACAACAGACCCACAC-3'; β-ACTIN forward: 5'-CCGCAAAGACCTGTACGCCAAC-3', reverse 5'-CCAGGGCAGTGATCTCCTTCTG-3'.

### Plasmids, shRNA constructs and viral production

The human IL8 promoter (bases -262 to -55) was amplified from MCF10A genomic DNA (DNeasy^® ^tissue kit, Qiagen) and cloned into pGL3 plasmid. pGL3-IL8 ΔκB and ΔE-box were generated by PCR site-directed mutagenesis (SDT). The coding sequences of TWIST1 full length and truncated (missing the last 20 amino acids, ΔWR) were amplified from pcDNA3-Tw [59] and subcloned into pcDNA4. SDT were induced to generate the C432A A433G mutations (S144R K145E) and C352T mutation (R118C). WT pcDNA4-TWIST1 was subcloned into pENTR4 (Invitrogen) to shuttle into pAd/CMV/V5-DEST (Invitrogen) by LR clonase II (Invitrogen). Adenoviral particles were packaged using HEK293A cells, according to the manufacturer's guidelines, and titrated. The mRNA target sequences of shRNAs shTw1 (shTw) and shTw2 were 5'-GGACAAGCUGAGCAAGAUU-3' and 5'-GCGACGAGCUGGACTCCAA-3', respectively. The sequences of shCtrl (mRNA target sequence UUCUCCGAACGUGUCACGU) and shIL8 (mRNA target sequence GCCAAGGAGUGCUAAAGAA) were previously reported [61]. shRNAs were created with siRNA sequences connected to a loop and a complementary sequence that were cloned into pcDNA3-U6 (gift from Dr. John Rossi, Beckman Research Institute of City of Hope). The U6-shRNA fragments were subcloned into pENTR4 (Invitrogen) to shuttle into pLenti6/Block-It™-DEST (Invitrogen). Lentiviral particles were generated, according to the manufacturer's guidelines, using HEK293FT cells. pGL3-3X κB, pCMV-IκBSR, pPCR-shGFP and pPCR-shRelA plasmids were gifts from Dr. Rama Natarajan (Beckman Research Institute of City of Hope).

### Luciferase assays

Cells were seeded in 24-well plates and co-transfected with pGL3 firefly luciferase promoter construct, pSV40-Renilla luciferase (Promega, San Luis Obispo, CA, USA) and transcription factor constructs of interest. After transfection (24 h), cell lysates were collected and firefly/renilla luciferase activities were assayed for luminescence using the Dual-Luciferase Reporter Assay System (Promega).

### Chromatin immunoprecipitation assay

ChIP assays were performed using the fast ChIP protocol [62] with minor modifications as follows. Cells were cross-linked on a dish with 1.1% formaldehyde (10 minutes, room temperature), quenched with 1.25 M glycine solution (5 minutes, room temperature), and collected in 1 ml PBS with protease inhibitors cocktail (Roche Applied Science Indianapolis, IN, USA)). Cells were then processed and IPs performed as described [62]. Precipitated chromatin was quantified using quantitative PCR and presented as percent of input. Anti-TWIST1 antibodies used for ChIP were custom-made to target the sequence GCQPPSGKRGGRKRRTSRRT. Anti-RELA antibodies were from Santa Cruz Biotechnology, Inc.; normal rabbit IgG was from Millipore (Temecula, CA, USA). Enrichment of IL8 promoter was analyzed by qPCR and presented by percent input using primers forward 5'-GTGATGACTCAGGTTTGCCC-3' and reverse 5'-GGTTGGTTTCTTCCTGGCTCT-3'. Control primers, forward 5'-ATCAGTCAAGCCAGGTTGTGTC-3', reverse 5'-AACACAGTGCATGGAGTGACAA-3', target a region 2 kb upstream of the transcription initiation site for the IL8 gene.

### Transwell migration/invasion assays

Transwell inserts (Millipore, 8 μm pore diameter) were pre-coated with 1 mg/ml fibronectin and equilibrated with serum free medium in a TC incubator for 1 h Cells (3.75 × 10^5 ^for MCF10A and MCF10ATw cells, 1 × 10^5 ^for BT549 cells) were resuspended in culture medium supplemented with 0.25% serum (400 μl) and loaded onto the upper well of inserts. Media (600 μl) that contained 20% serum and indicated components of interest were added to the lower well. For invasion assays, Matrigel (60 μl, 3 mg/ml, diluted with serum-free medium, BD Biosciences San Jose, California, USA) was layered on the upper membrane and placed in the TC incubator for 30 minutes to solidify. Cells were allowed to migrate/invade for 24 h, followed by fixation with 4% paraformaldehye and staining with hematoxilin and eosin. Transwell membranes were removed and cells on the upper side cleaned off with a cotton tip. Membranes were then mounted and images taken of the upper, lower, left, right and center of the membrane. Migrated or invaded cells were quantified using Image-ProPlus5.1 (Media Cybernetics, Inc. Rockville, MD 20850 USA). and data presented as a sum of the five images. Neutralizing anti-IL8 antibodies were purchased from R&D Systems; IL8 inhibitors repertaxin and SB225002 were from Sigma.

### Proliferation assays

Cells (3.75 × 10^4 ^for MCF10A and MCF10ATw cells; 10^4 ^for BT549 cells) were seeded in 96-well plates in equivalent conditions as for migration/invasion assays, and cell counts were determined with CellTiter 96 Aqueous One solution (Promega) according to the manufacturer's guidelines.

### Gelatin zymography

Conditioned media collected from MCF10A and MCF0ATw cells were filtered through a PVDF 0.45 μm low protein binding filter, concentrated using a 3000 NMWL Centricon concentrator (Millipore), mixed with β-mercaptoethanol-free loading buffer and resolved on a non-reducing PAGE gel that contained 0.1% (1 mg/ml) gelatin (Sigma). The gel was then incubated (1 h, room temperature, with shaking) in 2.5% Triton-X100 solution in water, followed by incubation (overnight, 37°C) in digestion buffer (10 mM calcium chloride, 20 mM Tris Acetic Acid, pH of 7.5). The gelatin gel was then stained with Coomassie Brilliant Blue (Bio-Rad) for 30 minutes at room temperature, washed with destaining solution (50:10:40 methanol:acetic acid:water) and imaged using a Kodak Electrophoresis Documentation and Analysis System 290 Eastman Kodak Company, Molecular Imaging Systems, New York, USA.

### Bioinformatics and statistical analysis

STOCKHOLM (GSE1456) and UPPSALA (GSE3494) microarray data sets were downloaded from the NCBI Gene Expression Omnibus. The data were divided into subtypes [33] based on information provided by Dr. Yudi Pawitan (Karolinska Institut, Sweden) and relative expression levels were represented by raw data. Non-parametric Mann-Whitney U test was performed to calculate the *P*-values presented.

## Abbreviations

bHLH: Basic helix-loop-helix; ChIP: chromatin immunoprecipitation; Co-IP: co-immunoprecipitation; EMT: epithelial-mesenchymal Transition; ECM: extracellular matrix; GSEA: Gene Set Enrichment Analysis; IL8: Interleukin 8; KD: knock-down; MMP: matrix metalloproteinase; NF-κB: Nuclear Factor-kappa B; SCS: Saethre-Chotzen Syndrome; shRNA: short hairpin RNA; TNF-α: Tumor Necrosis Factor alpha; WT: wild type

## Competing interests

The authors declare that they have no competing interests.

## Authors' contributions

SL carried out the molecular and cellular studies, participated in the experimental design and data analysis, and drafted the manuscript. SEK conceived of the study and carried out the cytokine array immunoblots. RR participated in the ChIP assays. JF carried out part of the ChIP assays. MC carried out the microarray study. ZL carried out the GSEA analysis and participated in the statistical analysis. GL participated in the qPCR and luciferase reporter assays. YHL, YHT, VL and SD participated in plasmid construction. SF participated in the microarray study and analysis. KSA participated in the initial design and coordination of this study and reviewed the manuscript. CG participated in conceiving of the study, its design and coordination, and helped to draft the manuscript. All authors read and approved the final manuscript.

## Supplementary Material

Additional file 1 **Figure S1. Cytokine pathway was enriched in MCF10ATw relative to MCF10A cells**. (**A**) Enrichment plot of changes in mRNA levels of cytokines included in the Biocarta_Cytokine_Pathway in MCF10ATw versus MCF10A cells. Profile of the running ES Score and positions of gene set members on the ranked list of genes are shown. (**B**) Heat map of cytokine expression in MCF10ATw versus MCF10A cells. Microarray data were performed in triplicate. Each row represents relative expression levels of a gene and each column represents one replicate of MCF10A or MCF10ATw cells. Red and blue indicate up-regulated or down-regulated expression, respectively. (**C**) Relative mRNA levels normalized to β-ACTIN (left) (See Methods) and secreted IL8 per 8 × 10^5 ^cells (right) from MCF10A and MCF10ATw cells. (**D**) Details of the GSEA analysis for the Biocarta_Cytokine_Pathway. Click here for file

Additional file 2 **Table S1**. Cytokine Array Map. Click here for file

Additional file 3 **Figure S2. IL8 promoter activation is independent of mutations in the TWIST1 bHLH DNA binding domain**. (**A**) Relative luciferase activities of SKBR3, MCF7 or BT549 cells 24 h post-transfection with IL8 WT or ΔE-box mutant promoter constructs together with TWIST1 or pcDNA vector control. WT, WT IL8 promoter; ΔE-box, ΔE-Box IL8 mutant promoter. (**B**) Relative luciferase activities of SKBR3 and MCF7 cells co-transfected with IL8 promoter reporter plasmid and WT, R118C, ΔWR, S144R K145E ΔWR or ΔbHLH (complete removal of the bHLH domain) mutant TWIST1. (**C**) Western blot of nuclear TWIST1 in SKBR3 and MCF7 cells that were transfected with or without TWIST1 and/or IκBSR. (**D**) Relative luciferase activities of IL8 promoter in BT549 cells stably transduced with shRNAs against TWIST1 and transiently transfected with either shGFP or shRelA (24 h). In A, B, D, data shown are from single representative experiments. Mean ± SD, n = 3, *P ≤ 0.05, ** *P *≤ 0.01. (**E**) Western blot of cytoplasmic and nuclear RELA in MCF10A and MCF10ATw cells. Cyto., cytoplasmic; nu., nuclear. (**F**) Immunoprecipitation of nuclear RELA in HEK293 cells that were transfected with indicated constructs. Normal rabbit IgG was used as controls. (**G**) ChIP assays using α-TWIST1 antibodies with solublized chromatin collected from HEK293 cells transfected with vector control, TWIST1, or TWIST1 and RELA for 48 h. Click here for file

## References

[B1] Sporn MB The war on cancer The Lancet 1996 347 1377 1381 10.1016/S0140-6736(96)91015-6 8637346

[B2] Yang J Mani SA Donaher JL Ramaswamy S Itzykson RA Come C Savagner P Gitelman I Richardson A Weinberg RA Twist, a master regulator of morphogenesis, plays an essential role in tumor metastasis Cell 2004 117 927 939 10.1016/j.cell.2004.06.006 15210113

[B3] Kwok WK Ling MT Lee TW Lau TC Zhou C Zhang X Chua CW Chan KW Chan FL Glackin C Wong YC Wang X Up-regulation of TWIST in prostate cancer and its implication as a therapeutic target Cancer Res 2005 65 5153 5162 10.1158/0008-5472.CAN-04-3785 15958559

[B4] Kajiyama H Hosono S Terauchi M Shibata K Ino K Yamamoto E Nomura S Nawa A Kikkawa F Twist expression predicts poor clinical outcome of patients with clear cell carcinoma of the ovary Oncology 2006 71 394 401 10.1159/000107108 17690559

[B5] Ou DL Chien HF Chen CL Lin TC Lin LI Role of Twist in head and neck carcinoma with lymph node metastasis Anticancer Res 2008 28 1355 1359 18505078

[B6] Matsuo N Shiraha H Fujikawa T Takaoka N Ueda N Tanaka S Nishina S Nakanishi Y Uemura M Takaki A Nakamura S Kobayashi Y Nouso K Yagi T Yamamoto K Twist expression promotes migration and invasion in hepatocellular carcinoma BMC Cancer 2009 9 240 10.1186/1471-2407-9-240 19615090 PMC2720986

[B7] Ru GQ Wang HJ Xu WJ Zhao ZS Upregulation of Twist in Gastric Carcinoma Associated with Tumor Invasion and Poor Prognosis Pathology Oncology Research 2010 1 7 10.1007/s12253-010-9332-0 21104359

[B8] Chen ZF Behringer RR twist is required in head mesenchyme for cranial neural tube morphogenesis Genes Dev 1995 9 686 699 10.1101/gad.9.6.686 7729687

[B9] Gitelman I Twist protein in mouse embryogenesis Dev Biol 1997 189 205 214 10.1006/dbio.1997.8614 9299114

[B10] Vesuna F van Diest P Chen JH Raman V Twist is a transcriptional repressor of E-cadherin gene expression in breast cancer Biochemical and Biophysical Research Communications 2008 367 235 241 10.1016/j.bbrc.2007.11.151 18062917 PMC2696127

[B11] Casas E Kim J Bendesky A Ohno-Machado L Wolfe CJ Yang J Snail2 is an essential mediator of Twist1-induced epithelial mesenchymal transition and metastasis Cancer Res 2011 71 245 254 10.1158/0008-5472.CAN-10-2330 21199805 PMC3025803

[B12] Kalluri R EMT: when epithelial cells decide to become mesenchymal-like cells J Clin Invest 2009 119 1417 1419 10.1172/JCI39675 19487817 PMC2689122

[B13] Chaffer CL Weinberg RA A perspective on cancer cell metastasis Science 2011 331 1559 1564 10.1126/science.1203543 21436443

[B14] Cheng GZ Chan J Wang Q Zhang W Sun CD Wang L-H Twist Transcriptionally Up-regulates AKT2 in Breast Cancer Cells Leading to Increased Migration, Invasion, and Resistance to Paclitaxel Cancer research 2007 67 1979 1987 10.1158/0008-5472.CAN-06-1479 17332325

[B15] Ma L Teruya-Feldstein J Weinberg RA Tumour invasion and metastasis initiated by microRNA-10b in breast cancer Nature 2007 449 682 688 10.1038/nature06174 17898713

[B16] Eckert Mark A Lwin Thinzar M Chang Andrew T Kim J Danis E Ohno-Machado L Yang J Twist1-Induced Invadopodia Formation Promotes Tumor Metastasis Cancer Cell 2011 19 372 386 10.1016/j.ccr.2011.01.036 21397860 PMC3072410

[B17] Castanon I Baylies MK A Twist in fate: evolutionary comparison of Twist structure and function Gene 2002 287 11 22 10.1016/S0378-1119(01)00893-9 11992718

[B18] El Ghouzzi V Legeai-Mallet L Benoist-Lasselin C Lajeunie E Renier D Munnich A Bonaventure J Mutations in the basic domain and the loop-helix II junction of TWIST abolish DNA binding in Saethre-Chotzen syndrome FEBS Lett 2001 492 112 118 10.1016/S0014-5793(01)02238-4 11248247

[B19] Gripp KW Zackai EH Stolle CA Mutations in the human TWIST gene Human Mutation 2000 15 150 155 10.1002/(SICI)1098-1004(200002)15:2<150::AID-HUMU3>3.0.CO;2-D 10649491

[B20] Howard TD Paznekas WA Green ED Chiang LC Ma N Ortiz de Luna RI Garcia Delgado C Gonzalez-Ramos M Kline AD Jabs EW Mutations in TWIST, a basic helix-loop-helix transcription factor, in Saethre-Chotzen syndrome Nat Genet 1997 15 36 41 10.1038/ng0197-36 8988166

[B21] Bialek P Kern B Yang X Schrock M Sosic D Hong N Wu H Yu K Ornitz DM Olson EN Justice MJ Karsenty G A Twist Code Determines the Onset of Osteoblast Differentiation Developmental Cell 2004 6 423 435 10.1016/S1534-5807(04)00058-9 15030764

[B22] Subramanian A Tamayo P Mootha VK Mukherjee S Ebert BL Gillette MA Paulovich A Pomeroy SL Golub TR Lander ES Mesirov JP Gene set enrichment analysis: a knowledge-based approach for interpreting genome-wide expression profiles Proc Natl Acad Sci USA 2005 102 15545 15550 10.1073/pnas.0506580102 16199517 PMC1239896

[B23] Liu M Ju X Willmarth NE Casimiro MC Ojeifo J Sakamaki T Katiyar S Jiao X Popov VM Yu Z Wu K Joyce D Wang C Pestell RG Nuclear factor-kappaB enhances ErbB2-induced mammary tumorigenesis and neoangiogenesis in vivo Am J Pathol 2009 174 1910 1920 10.2353/ajpath.2009.080706 19349372 PMC2671278

[B24] Mukaida N Mahe Y Matsushima K Cooperative interaction of nuclear factor-kappa B- and cis-regulatory enhancer binding protein-like factor binding elements in activating the interleukin-8 gene by pro-inflammatory cytokines J Biol Chem 1990 265 21128 21133 2250017

[B25] Stein B Baldwin AS Jr Distinct mechanisms for regulation of the interleukin-8 gene involve synergism and cooperativity between C/EBP and NF-kappa B Mol Cell Biol 1993 13 7191 7198 8413306 10.1128/mcb.13.11.7191-7198.1993 PMC364780

[B26] Sosic D Richardson JA Yu K Ornitz DM Olson EN Twist regulates cytokine gene expression through a negative feedback loop that represses NF-kappaB activity Cell 2003 112 169 180 10.1016/S0092-8674(03)00002-3 12553906

[B27] Traenckner EB Pahl HL Henkel T Schmidt KN Wilk S Baeuerle PA Phosphorylation of human I kappa B-alpha on serines 32 and 36 controls I kappa B-alpha proteolysis and NF-kappa B activation in response to diverse stimuli EMBO J 1995 14 2876 2883 7796813 10.1002/j.1460-2075.1995.tb07287.x PMC398406

[B28] Shirokawa JM Courey AJ A direct contact between the dorsal rel homology domain and Twist may mediate transcriptional synergy Mol Cell Biol 1997 17 3345 3355 9154833 10.1128/mcb.17.6.3345 PMC232187

[B29] Chakrabarti S Patel KD Regulation of matrix metalloproteinase-9 release from IL-8-stimulated human neutrophils J Leukoc Biol 2005 78 279 288 10.1189/jlb.1004612 15831558

[B30] Li A Dubey S Varney ML Dave BJ Singh RK IL-8 directly enhanced endothelial cell survival, proliferation, and matrix metalloproteinases production and regulated angiogenesis J Immunol 2003 170 3369 3376 12626597 10.4049/jimmunol.170.6.3369

[B31] White JR Lee JM Young PR Hertzberg RP Jurewicz AJ Chaikin MA Widdowson K Foley JJ Martin LD Griswold DE Sarau HM Identification of a potent, selective non-peptide CXCR2 antagonist that inhibits interleukin-8-induced neutrophil migration J Biol Chem 1998 273 10095 10098 10.1074/jbc.273.17.10095 9553055

[B32] Casilli F Bianchini A Gloaguen I Biordi L Alesse E Festuccia C Cavalieri B Strippoli R Cervellera MN Di Bitondo R Ferretti E Mainiero F Bizzarri C Colotta F Bertini R Inhibition of interleukin-8 (CXCL8/IL-8) responses by repertaxin, a new inhibitor of the chemokine receptors CXCR1 and CXCR2 Biochem Pharmacol 2005 69 385 394 10.1016/j.bcp.2004.10.007 15652230

[B33] Calza S Hall P Auer G Bjohle J Klaar S Kronenwett U Liu ET Miller L Ploner A Smeds J Bergh J Pawitan Y Intrinsic molecular signature of breast cancer in a population-based cohort of 412 patients Breast Cancer Res 2006 8 R34 10.1186/bcr1517 16846532 PMC1779468

[B34] Hamamori Y Sartorelli V Ogryzko V Puri PL Wu HY Wang JY Nakatani Y Kedes L Regulation of histone acetyltransferases p300 and PCAF by the bHLH protein twist and adenoviral oncoprotein E1A Cell 1999 96 405 413 10.1016/S0092-8674(00)80553-X 10025406

[B35] Hamamori Y Wu HY Sartorelli V Kedes L The basic domain of myogenic basic helix-loop-helix (bHLH) proteins is the novel target for direct inhibition by another bHLH protein, Twist Mol Cell Biol 1997 17 6563 6573 9343420 10.1128/mcb.17.11.6563 PMC232510

[B36] Ghosh S May MJ Kopp EB NF-kappa B and Rel proteins: evolutionarily conserved mediators of immune responses Annu Rev Immunol 1998 16 225 260 10.1146/annurev.immunol.16.1.225 9597130

[B37] Baker RG Hayden MS Ghosh S NF-[kappa]B, Inflammation, and Metabolic Disease Cell Metabolism 2011 13 11 22 10.1016/j.cmet.2010.12.008 21195345 PMC3040418

[B38] Ben-Neriah Y Karin M Inflammation meets cancer, with NF-[kappa]B as the matchmaker Nat Immunol 2011 12 715 723 21772280 10.1038/ni.2060

[B39] Bollrath J Greten FR IKK/NF-[kappa]B and STAT3 pathways: central signalling hubs in inflammation-mediated tumour promotion and metastasis EMBO Rep 2009 10 1314 1319 10.1038/embor.2009.243 19893576 PMC2799209

[B40] Jiang J Kosman D Ip YT Levine M The dorsal morphogen gradient regulates the mesoderm determinant twist in early Drosophila embryos Genes & Development 1991 5 1881 1891 10.1101/gad.5.10.1881 1655572

[B41] Ip YT Park RE Kosman D Yazdanbakhsh K Levine M dorsal-twist interactions establish snail expression in the presumptive mesoderm of the Drosophila embryo Genes Dev 1992 6 1518 1530 10.1101/gad.6.8.1518 1644293

[B42] Szymanski P Levine M Multiple modes of dorsal-bHLH transcriptional synergy in the Drosophila embryo EMBO J 1995 14 2229 2238 7774581 10.1002/j.1460-2075.1995.tb07217.x PMC398329

[B43] Pham CG Bubici C Zazzeroni F Knabb JR Papa S Kuntzen C Franzoso G Upregulation of Twist-1 by NF-κB Blocks Cytotoxicity Induced by Chemotherapeutic Drugs Molecular and Cellular Biology 2007 27 3920 3935 10.1128/MCB.01219-06 17403902 PMC1900008

[B44] Derin D Soydinc HO Guney N Tas F Camlica H Duranyildiz D Yasasever V Topuz E Serum IL-8 and IL-12 levels in breast cancer Med Oncol 2007 24 163 168 10.1007/BF02698035 17848739

[B45] De Larco JE Wuertz BR Rosner KA Erickson SA Gamache DE Manivel JC Furcht LT A potential role for interleukin-8 in the metastatic phenotype of breast carcinoma cells Am J Pathol 2001 158 639 646 10.1016/S0002-9440(10)64005-9 11159200 PMC1850317

[B46] Green AR Green VL White MC Speirs V Expression of cytokine messenger RNA in normal and neoplastic human breast tissue: identification of interleukin-8 as a potential regulatory factor in breast tumours Int J Cancer 1997 72 937 941 10.1002/(SICI)1097-0215(19970917)72:6<937::AID-IJC3>3.0.CO;2-Q 9378554

[B47] Benoy IH Salgado R Van Dam P Geboers K Van Marck E Scharpé S Vermeulen PB Dirix LY Increased Serum Interleukin-8 in Patients with Early and Metastatic Breast Cancer Correlates with Early Dissemination and Survival Clinical Cancer Research 2004 10 7157 7162 10.1158/1078-0432.CCR-04-0812 15534087

[B48] Orditura M De Vita F Catalano G Infusino S Lieto E Martinelli E Morgillo F Castellano P Pignatelli C Galizia G Elevated serum levels of interleukin-8 in advanced non-small cell lung cancer patients: relationship with prognosis J Interferon Cytokine Res 2002 22 1129 1135 10.1089/10799900260442557 12513912

[B49] Waugh DJ Wilson C The interleukin-8 pathway in cancer Clin Cancer Res 2008 14 6735 6741 10.1158/1078-0432.CCR-07-4843 18980965

[B50] Baggiolini M Walz A Kunkel SL Neutrophil-activating peptide-1/interleukin 8, a novel cytokine that activates neutrophils J Clin Invest 1989 84 1045 1049 10.1172/JCI114265 2677047 PMC329758

[B51] Hunninghake GW Gadek JE Lawley TJ Crystal RG Mechanisms of neutrophil accumulation in the lungs of patients with idiopathic pulmonary fibrosis J Clin Invest 1981 68 259 269 10.1172/JCI110242 7251862 PMC370793

[B52] Idell S Kucich U Fein A Kueppers F James HL Walsh PN Weinbaum G Colman RW Cohen AB Neutrophil elastase-releasing factors in bronchoalveolar lavage from patients with adult respiratory distress syndrome Am Rev Respir Dis 1985 132 1098 1105 3877483 10.1164/arrd.1985.132.5.1098

[B53] Rola-Pleszczynski M Gouin S Begin R Asbestos-induced lung inflammation. Role of local macrophage-derived chemotactic factors in accumulation of neutrophils in the lungs Inflammation 1984 8 53 62 10.1007/BF00918353 6715031

[B54] Schmidt KG Rasmussen JW Wedebye IM Frederiksen PB Pedersen NT Accumulation of Indium-111-Labeled Granulocytes in Malignant Tumors Journal of Nuclear Medicine 1988 29 479 484 3351603

[B55] Fridlender ZG Sun J Kim S Kapoor V Cheng G Ling L Worthen GS Albelda SM Polarization of tumor-associated neutrophil phenotype by TGF-beta: "N1" versus "N2" TAN Cancer Cell 2009 16 183 194 10.1016/j.ccr.2009.06.017 19732719 PMC2754404

[B56] Koch AE Polverini PJ Kunkel SL Harlow LA DiPietro LA Elner VM Elner SG Strieter RM Interleukin-8 as a macrophage-derived mediator of angiogenesis Science 1992 258 1798 1801 10.1126/science.1281554 1281554

[B57] Mironchik Y Winnard PT Vesuna F Kato Y Wildes F Pathak AP Kominsky S Artemov D Bhujwalla Z Van Diest P Burger H Glackin C Raman V Twist Overexpression Induces In vivo Angiogenesis and Correlates with Chromosomal Instability in Breast Cancer Cancer research 2005 65 10801 10809 10.1158/0008-5472.CAN-05-0712 16322226 PMC5575828

[B58] Vesuna F Lisok A Kimble B Raman V Twist modulates breast cancer stem cells by transcriptional regulation of CD24 expression Neoplasia 2009 11 1318 1328 20019840 10.1593/neo.91084 PMC2794513

[B59] Lee MS Lowe GN Strong DD Wergedal JE Glackin CA TWIST, a basic helix-loop-helix transcription factor, can regulate the human osteogenic lineage J Cell Biochem 1999 75 566 577 10.1002/(SICI)1097-4644(19991215)75:4<566::AID-JCB3>3.0.CO;2-0 10572240

[B60] Debnath J Muthuswamy SK Brugge JS Morphogenesis and oncogenesis of MCF-10A mammary epithelial acini grown in three-dimensional basement membrane cultures Methods 2003 30 256 268 10.1016/S1046-2023(03)00032-X 12798140

[B61] Merritt WM Lin YG Spannuth WA Fletcher MS Kamat AA Han LY Landen CN Jennings N De Geest K Langley RR Villares G Sanguino A Lutgendorf SK Lopez-Berestein G Bar-Eli MM Sood AK Effect of interleukin-8 gene silencing with liposome-encapsulated small interfering RNA on ovarian cancer cell growth J Natl Cancer Inst 2008 100 359 372 10.1093/jnci/djn024 18314475 PMC2770251

[B62] Nelson JD Denisenko O Bomsztyk K Protocol for the fast chromatin immunoprecipitation (ChIP) method Nat Protoc 2006 1 179 185 10.1038/nprot.2006.27 17406230

