# Supplementary figures and images for: TWIST1 associates with NF-κB subunit RELA via carboxyl-terminal WR domain to promote cell autonomous invasion through IL8 production
Source: BMC Biol. 2012 Aug 14;10:73. doi: 10.1186/1741-7007-10-73 (PMC3482588; doi:10.1186/1741-7007-10-73)

Additional File 1

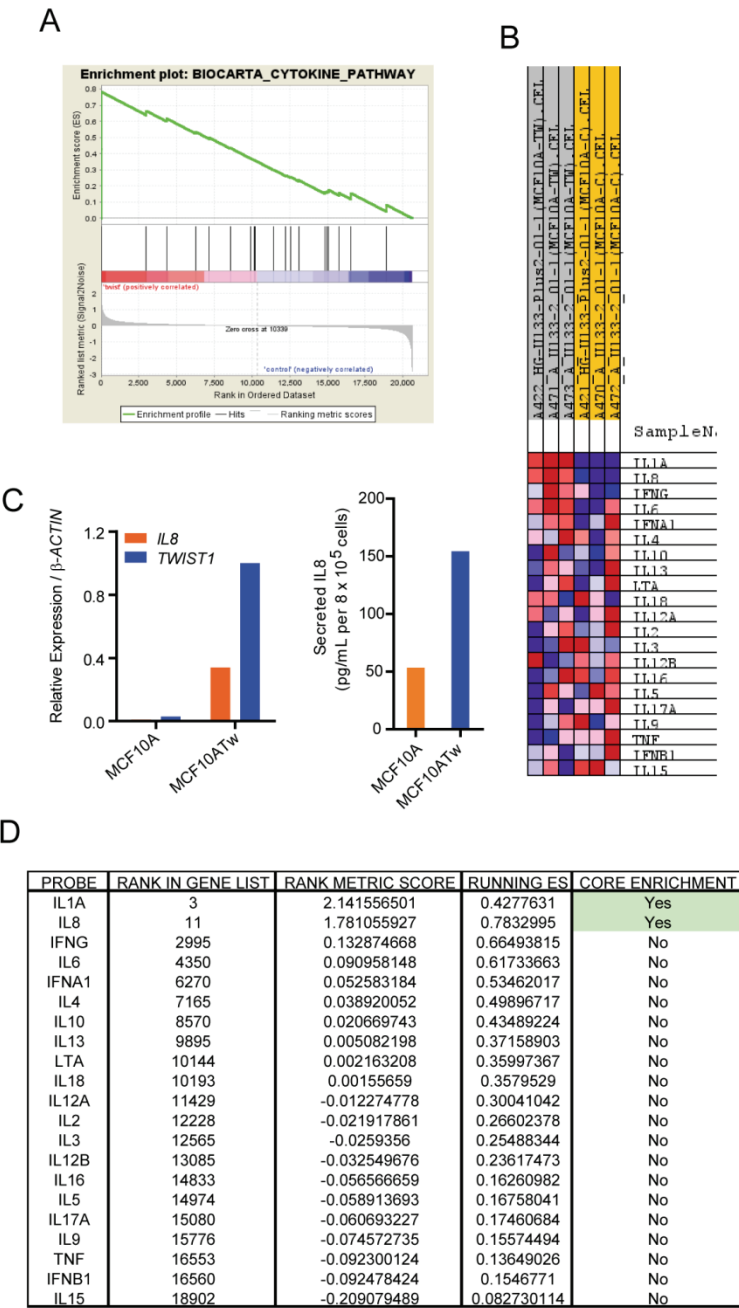

Figure. S1

Supplement: Additional file 1 — Figure S1. Cytokine pathway was enriched in MCF10ATw relative to MCF10A cells. (A) Enrichment plot of changes in mRNA levels of cytokines included in the Biocarta_Cytokine_Pathway in MCF10ATw versus MCF10A cells. Profile of the running ES Score and positions of gene set members on the ranked list of genes are shown. (B) Heat map of cytokine expression in MCF10ATw versus MCF10A cells. Microarray data were performed in triplicate. Each row represents relative expression levels of a gene and each column represents one replicate of MCF10A or MCF10ATw cells. Red and blue indicate up-regulated or down-regulated expression, respectively. (C) Relative mRNA levels normalized to β-ACTIN (left) (See Methods) and secreted IL8 per 8 × 10^5^ cells (right) from MCF10A and MCF10ATw cells. (D) Details of the GSEA analysis for the Biocarta_Cytokine_Pathway. [file 1741-7007-10-73-S1.PDF]

## Additional File 3

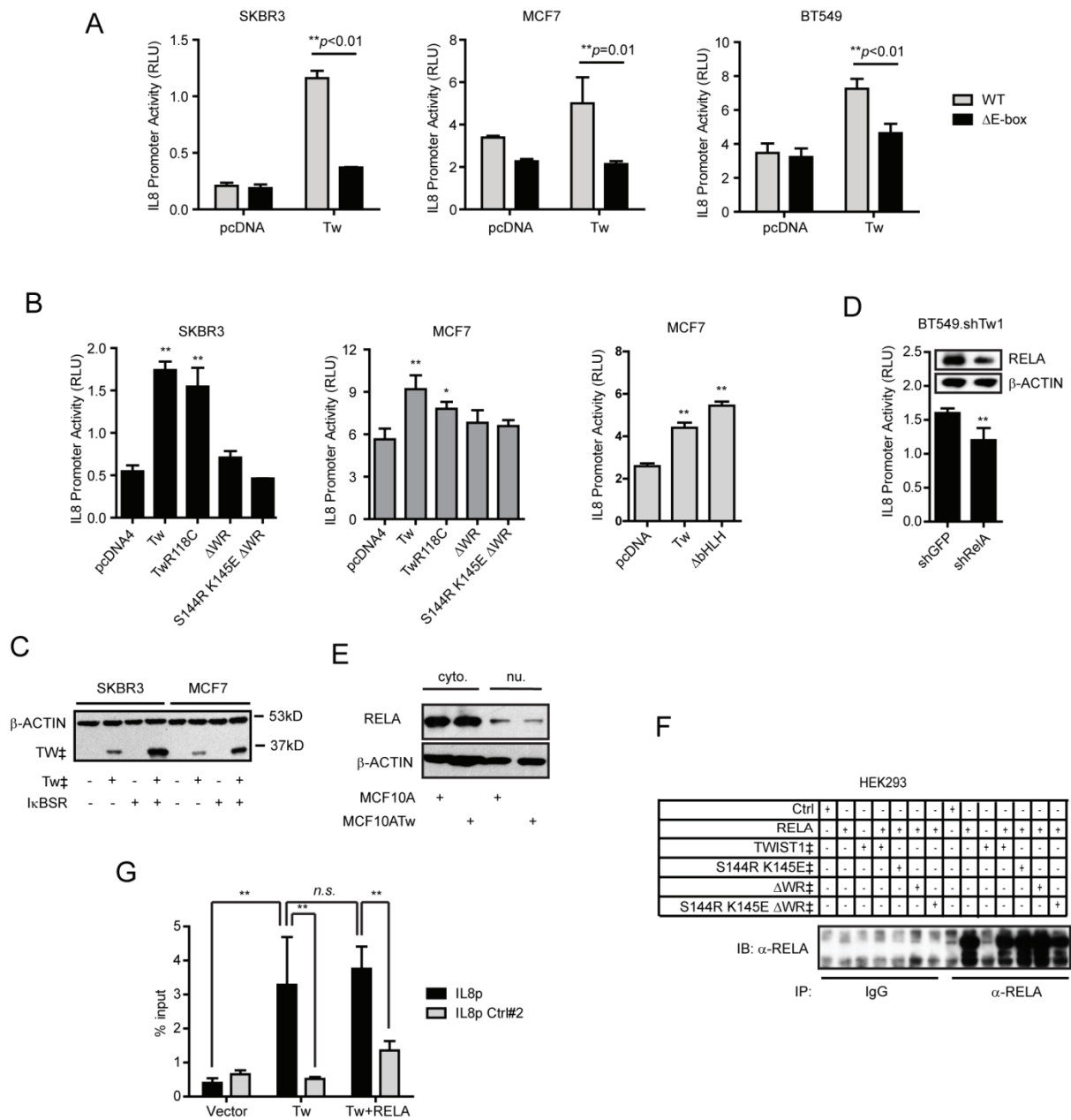

Figure. S2

Supplement: Additional file 3 — Figure S2. IL8 promoter activation is independent of mutations in the TWIST1 bHLH DNA binding domain. (A) Relative luciferase activities of SKBR3, MCF7 or BT549 cells 24 h post-transfection with IL8 WT or ΔE-box mutant promoter constructs together with TWIST1 or pcDNA vector control. WT, WT IL8 promoter; ΔE-box, ΔE-Box IL8 mutant promoter. (B) Relative luciferase activities of SKBR3 and MCF7 cells co-transfected with IL8 promoter reporter plasmid and WT, R118C, ΔWR, S144R K145E ΔWR or ΔbHLH (complete removal of the bHLH domain) mutant TWIST1. (C) Western blot of nuclear TWIST1 in SKBR3 and MCF7 cells that were transfected with or without TWIST1 and/or IκBSR. (D) Relative luciferase activities of IL8 promoter in BT549 cells stably transduced with shRNAs against TWIST1 and transiently transfected with either shGFP or shRelA (24 h). In A, B, D, data shown are from single representative experiments. Mean ± SD, n = 3, *P ≤ 0.05, ** P ≤ 0.01. (E) Western blot of cytoplasmic and nuclear RELA in MCF10A and MCF10ATw cells. Cyto., cytoplasmic; nu., nuclear. (F) Immunoprecipitation of nuclear RELA in HEK293 cells that were transfected with indicated constructs. Normal rabbit IgG was used as controls. (G) ChIP assays using α-TWIST1 antibodies with solublized chromatin collected from HEK293 cells transfected with vector control, TWIST1, or TWIST1 and RELA for 48 h. [file 1741-7007-10-73-S3.PDF]
