# Supplementary material for: TWIST1 associates with NF-κB subunit RELA via carboxyl-terminal WR domain to promote cell autonomous invasion through IL8 production
Source: BMC Biol. 2012 Aug 14;10:73. doi: 10.1186/1741-7007-10-73 (PMC3482588; doi:10.1186/1741-7007-10-73)
Supplement: Additional file 2 — Table S1. Cytokine Array Map. [file 1741-7007-10-73-S2.PDF]

Additional File 2

Table S1. Cytokine Array Map

Pos	Pos	Neg	Neg	EMA-78	GCSF	GM-CSF	GRO	GRO-α	I-309	IL-1α	IL-1β
Pos	Pos	Neg	Neg	EMA-78	GCSF	GM-CSF	GRO	GRO-α	I-309	IL-1α	IL-1β
IL-2	IL-3	IL-4	IL-5	IL-6	IL-7	IL-8	IL-10	IL-12	IL-13	IL-15	IFN-γ
IL-2	IL-3	IL-4	IL-5	IL-6	IL-7	IL-8	IL-10	IL-12	IL-13	IL-15	IFN-γ
MCP-1	MCP-2	MCP-3	MCSF	MDC	MIG	MIP-1β	RANTES	SCF	SDF-1	TARC	TGF-β1
MCP-1	MCP-2	MCP-3	MCSF	MDC	MIG	MIP-1β	RANTES	SCF	SDF-1	TARC	TGF-β1
TNF-α	TNF-β	EGF	IGF-1	Ang	OSM	THPO	VEGF	PDGF-β	LEP	Neg	Pos
TNF-α	TNF-β	EGF	IGF-1	Ang	OSM	THPO	VEGF	PDGF-β	LEP	Neg	Pos
